# Ten quick tips for getting the most scientific value out of numerical data

**DOI:** 10.1371/journal.pcbi.1006141

**Published:** 2018-10-11

**Authors:** Lars Ole Schwen, Sabrina Rueschenbaum

**Affiliations:** 1 Fraunhofer MEVIS, Am Fallturm 1, Bremen, Germany; 2 Department of Internal Medicine 1, University Hospital Frankfurt, Goethe University, Theodor-Stern-Kai 7, Frankfurt (Main), Germany; Genome Quebec, CANADA

## Abstract

Most studies in the life sciences and other disciplines involve generating and analyzing numerical data of some type as the foundation for scientific findings. Working with numerical data involves multiple challenges. These include reproducible data acquisition, appropriate data storage, computationally correct data analysis, appropriate reporting and presentation of the results, and suitable data interpretation.

Finding and correcting mistakes when analyzing and interpreting data can be frustrating and time-consuming. Presenting or publishing incorrect results is embarrassing but not uncommon. Particular sources of errors are inappropriate use of statistical methods and incorrect interpretation of data by software. To detect mistakes as early as possible, one should frequently check intermediate and final results for plausibility. Clearly documenting how quantities and results were obtained facilitates correcting mistakes. Properly understanding data is indispensable for reaching well-founded conclusions from experimental results. Units are needed to make sense of numbers, and uncertainty should be estimated to know how meaningful results are. Descriptive statistics and significance testing are useful tools for interpreting numerical results if applied correctly. However, blindly trusting in computed numbers can also be misleading, so it is worth thinking about how data should be summarized quantitatively to properly answer the question at hand. Finally, a suitable form of presentation is needed so that the data can properly support the interpretation and findings. By additionally sharing the relevant data, others can access, understand, and ultimately make use of the results.

These quick tips are intended to provide guidelines for correctly interpreting, efficiently analyzing, and presenting numerical data in a useful way.

## Author summary

Data expressed as numbers are ubiquitous in research in the life sciences and other fields. The typical scientific workflow using such numerical data consists of analyzing the raw data to obtain numerical results, followed by interpreting the results and presenting derived findings.

In this article, we present some tips and tricks for working with numerical data. We discuss how data can be checked effectively for plausibility—and that this should be done frequently to spot potential mistakes early. Keeping clear records of the way raw numbers and intermediate or final results were obtained helps correct mistakes efficiently. To facilitate correct interpretation of data, we suggest paying attention to correct units of values and to understandable formulas. Appropriate statistical methods should be used for the specific question at hand. To avoid an over-interpretation of numbers, uncertainty of numbers should be respected, and one should be aware of cases in which bare numbers are actually misleading. Finally, we provide some recommendations for presenting data in a way useful for supporting interpretation and findings, ideally so that follow-up work can make use of the data.

## Introduction

### Background

**Fig 1:**
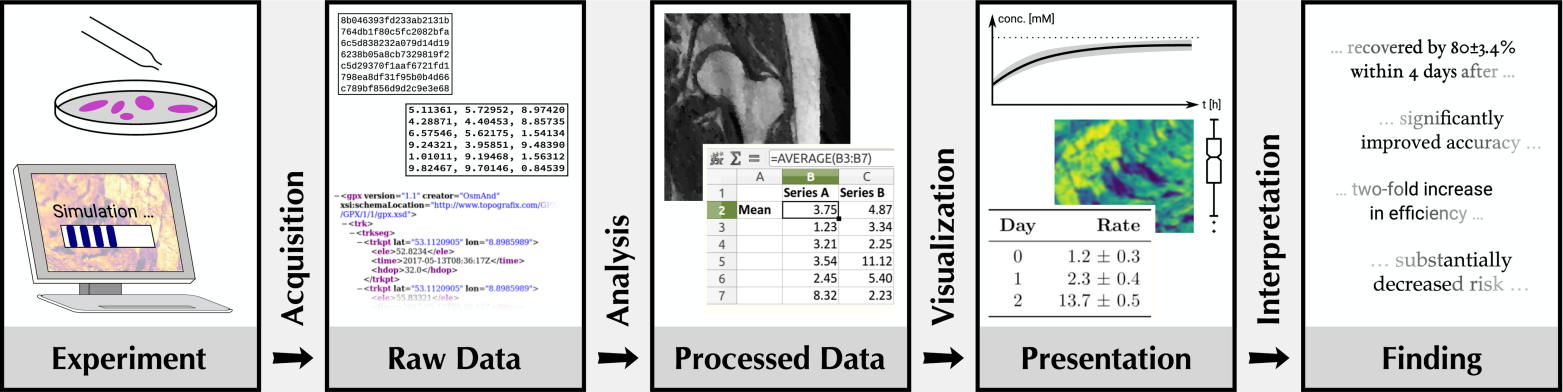
Typical steps in scientific work involving data analysis.

In many studies in the life sciences and other fields, findings are derived from some type of experiment via the analysis of numerical data in one form or another. The typical workflow, cf. Fig 1, involves multiple steps where good scientific practice needs to be followed so that sound results are obtained.

Experiments (regardless of whether they are in vivo, in vitro, or in silico) need to be designed and documented so that data of interest can be acquired reproducibly [1]. Data need to be stored in a reliable way that allows efficient finding and proper use [2]. Data processing and analysis need to be correct and reproducible in order to obtain meaningful results [3]. In particular, statistical analyses need to be done correctly [4]. Data need to be presented in an understandable form to show results and to support the conclusions drawn from it, which is typically achieved by presenting data in graphical form [5]. There are, however, many pitfalls when working with numerical data in these steps.

Mistakes when analyzing numerical data lead to incorrect results and incorrect interpretations. Correcting such mistakes can be time-consuming, depending on how much follow-up effort you or others have already spent, and realizing that one has wasted effort is usually frustrating. Not realizing mistakes yourself and presenting incorrect results to others can be rather embarrassing, in particular when the false results have already been published. Sources of errors in publications include but are not limited to applying inappropriate statistical methods [6] and incorrect interpretation of data by software [7]. Unfortunately, there is no way to completely avoid mistakes when working with data. (If you do know one, please let us know!) However, knowing about potential pitfalls, one can at least reduce the number of mistakes.

Properly understanding the data you are working with is probably the most important aspect when trying to avoid mistakes. Frequently asking yourself, “Does this make sense?” helps you spot mistakes early and avoids realizing at some later point, “Why didn't I see this stupid mistake earlier?!” Understanding the data at hand is also necessary to distinguish relevant effects in observed data from meaningless effects so that you can focus research and analysis efforts on interesting investigations to obtain valuable results.

### Motivation

The purpose of this article is to provide some guidelines for working with numerical data for researchers such as biologists, wet-lab experimentalists, computational scientists, and data scientists. We present a number of tips on how to make understanding numerical data easier, how to correctly work with it, and how to present results in a useful way to others. “Others” in this context might be a wide range of persons whom you might prefer not to annoy, e.g., a reader or reviewer of your research paper, your advisor grading your thesis, or your favorite colleague who will build on your work. This "other" person might also be yourself following up on previous work a few months from now.

The examples we chose for illustrating these guidelines range from rather generic applications to specific biological questions. Clearly, the effects we mean to point out by such examples may also occur more generally in various other contexts.

### Outline

The article is structured in tips about how to keep data correct (Tips 1 to 3), how to correctly interpret it (Tips 4 to 8), and how to present it in a useful and nonambiguous form (Tips 9 and 10).

## Keep your data correct

### Tip 1: Check your data for plausibility

Frequent plausibility checking is probably the most useful approach to quickly spot mistakes—when acquiring data, in computations, and when interpreting results. Whenever you realize a mistake and you get the feeling you could have found it already much earlier, this can be a sign of too infrequent plausibility checking. Several techniques can help with plausibility checking.

#### Searching for surprising patterns

A simple yet effective technique is to look for unexpected patterns in data and to think about whether there is a plausible explanation after all or whether you need to go back one or more steps and correct some mistake. For instance, if two out of every seven values in the weight recordings of your lab animals are zero, this might indicate daily measurements not performed on weekends and erroneously interpreted as zero. Another pattern to beware of is numbers or their differences being equal or surprisingly round. For instance, when counting colony-forming units of *Escherichia coli* on agar plates, obtaining 15, 46, 28, 46, 52, and 46 colonies contains suspiciously many repeated values, indicating that numbers might have been mixed up. Or, suppose your data contains time points of *t*_1_ = 16421.392 s and *t*_2_ = 20021.392 s; there may be a perfectly valid explanation for this offset of 3,600 seconds (you are measuring something exactly every hour). However, this might also indicate that *t*_2_ was not measured properly but accidentally computed from *t*_1_ with a fixed increment. Besides assessing the data all by yourself, it can also be useful to look at the data together with a colleague and discuss the meaning of observed values and patterns. Another person might notice issues you missed before.

#### Guesstimation

Another very useful technique is to combine guesstimation and back-of-the-envelope calculations, i.e., to make simplified assumptions and estimates (e.g., guesses about orders of magnitude) and use approximate and short calculations (see, e.g., [8]). This can also be useful in everyday life: If you want to fly from Toronto, Ontario (Canada) to Sydney, New South Wales (Australia) and you are offered a flight taking 2.5 hours, estimating the distance (other side of the planet, i.e., more than 10,000 km) and the speed of airplanes (below 1,000 km/h, i.e., the flight should take at least 10 hours) can prevent you from ending up in Sydney, Nova Scotia (Canada). For further (rather amusing) guesstimates, we refer to [9].

Guesstimation can generally be applied in two ways—retrospectively (checking results you have already obtained) and prospectively (estimating results that are not available yet). Retrospective guesstimation is useful to check for plausibility (unfortunately not to prove correctness, though). Prospective guesstimation can be applied to estimate costs and efforts of planned experiments or to give an idea in which order of magnitude to expect results you have not obtained yet. However, this should be done and communicated with care to avoid observers being biased by "expected" results.

As an example for checking plausibility by guesstimation, suppose you read a study about the growth of newborn Humboldt penguins (*Spheniscus humboldti*), reporting that they grew 1/4 inch per day on average during one year. Is this plausible? One week has about 8 days (i.e., 2 inches per week), one year has about 50 weeks (i.e., 100 inches in one year), one inch is about 2.5 cm (i.e., 250 cm in one year), and one-year-old penguins are nowhere near 2.5 meters tall. So this is not plausible.

As an example for prospective guesstimation, suppose you want to assess the functionality of the nonstructural protein 3 (NS3) from Zika virus by protein crystallization, which requires a yield of 20 mg of protein. You need to express NS3 in bacteria, without reaching cytotoxic protein concentrations in the bacterial culture, and purify it afterwards. Is the incubator in your lab big enough for this experiment? From your colleague's experience with the NS3 protein from hepatitis C virus, which shares protein function and viral family, you estimate that a yield of 2 mg per liter bacterial culture is possible without cytotoxic effects. Therefore, you need at least 20/2 = 10 liters of bacterial culture.

### Tip 2: Track your sources

Numerical results are typically based on literature data or on data acquired from your own experiments. In any scientific work, it is crucial to document in sufficient detail how these data were acquired and which calculations were performed so that the reported results can be reproduced. Below, we will discuss working with literature data and making calculations reproducible. For recommendations on how to document data acquisition in wet lab and in silico experiments, we refer to [10] and [1], respectively.

#### Literature data

When using data from the literature, it is useful to immediately write down a unique identifier of the source. For this purpose, the DOI of the manuscript, a full reference, or the identifier used in your literature management are better suited than something unspecific like "the snail paper by Smith," which might not be clear to you anymore in a few weeks. Besides writing down where a number comes from, it is helpful to separate any conversion you used from the exact number in the source, e.g., noting that your constant *v*_mean_ = 1.27 millimeters per second was written as *V*_average_ = 3 inches per minute in the source so that you can easily search for it again.

#### Automated data analysis

Analysis of raw numerical data typically requires one or more steps of computation before reaching some conclusion, cf. Fig 1. It is very useful to be able to run these computations in an automated and reproducible way, ideally in a one-step procedure (one click, a single script, or a similar technique). Performing the computations once is usually not sufficient. There will typically be additional measurements at some point, mistakes in the analysis workflow to be fixed, or requests (e.g., by reviewers or your advisor) to add similar and closely related analyses. This is "like a version of Murphy's Law: Everything you do, you will probably have to do over again" [11]. It makes sense to also include visualization and/or plotting in this one-step procedure, as you will probably have to repeatedly redo graphics with slight changes before you (and maybe your co-authors, advisor, or reviewers) are satisfied with the result. Besides offering the chance of easily adapting analysis and visualization, this also precisely documents how results and figures were obtained without you needing to remember technical details (“How thick was this line again? 0.8 or 1 points?” or “How did I make the axis labels appear rotated?” etc.).

#### Separation of data and formulas

For automated data analysis, it is preferable to separate data from formulas via scripting (R, Python, or your favorite language), rather than to keep data and formulas in spreadsheets. (We chose Python for the data analysis provided in the supporting information. For an introductory overview for other choices of programming languages for computational biology, we refer to [12].) This simplifies testing computations by checking output for simple input data and by checking whether varying input data has the expected or at least plausible impact on final results. Moreover, it reduces the risk of mistakes when updating data: for instance, suppose your spreadsheet computes mean and standard deviation of 284 values, but the data need to be updated. Pasting new data with only 273 values might not overwrite all old data; pasting new data with 298 values means that cell ranges in the formulas computing mean and standard deviation need to be updated. Both are easy to miss and likely to go unnoticed.

Moreover, applying basic techniques from software development to the data analysis can be useful. All evaluation methods should be tested, ideally by writing the test before implementing the computation (using so-called test-driven development [13]). Implementation of the data analysis should be well structured, documented and kept under revision control. This additionally facilitates cooperation between multiple contributors (or one person working at different computers). Revison control ensures that there is a clearly identified authoritative latest version of data, evaluation, and results and easily provides backups of previous versions of your evaluation. Also for manuscripts, revision control is useful. For further recommendations for scientific computing, we refer to [14, 15]. Besides testing individual steps of the data analysis for correctness, intermediate and final results from the entire workflow should be checked for plausibility (cf. Tip 1).

### Tip 3: Beware of computational pitfalls

In order to get the implementation of data analysis right, it is necessary to know capabilities, limitations, and pitfalls of whatever tools you are using. Calculators require manual data input, which is error-prone and not automatic; software tools (Excel, R, Python, etc.) have their own peculiarities and possibly surprising behavior, e.g., in case of missing data or user input. In this tip, we discuss a few generic pitfalls without focusing on specific software.

#### Using copy and paste

Copying and pasting intermediate results in a calculation workflow is generally a bad idea because this will not automatically update if input data changes. Also, copying values might introduce unnecessary premature rounding of numbers, in particular, if the values are read and typed rather than copied and pasted.

#### Integer division

Division of numbers may, in some cases, be interpreted as integer division with remainder. As an example, one of the authors (LOS) recently tried computing a success percentage by dividing the number of acceptable cases by the number of all cases, which results, e.g., in 84/96 = 0. This was detected only aft…, erm, without any delay, of course, by thorough testing and immediate plausibility checking.

#### Unavailable data points

In structured measurements and data sets, not all data points are necessarily available (e.g., certain time points of periodic measurements may be missing). Such unavailable data need to be treated correctly. Silently interpreting it as zero or counting data points regardless of whether they are available may lead to wrong results. Even worse, these results may still look plausible if only few data points are unavailable.

#### Floating-point numbers and nonfinite values

When computing noninteger results, most software uses floating-point numbers (floats) [16]. Even though this may seem like an internal detail, it is worth knowing that this may result in artifacts and surprising results.

On the one hand, rounding effects may occur because floats only have finite precision and cannot represent every fractional number exactly (not even a simple 0.1). On the other hand, floats can deal with division 0.0/0.0 or other illegal mathematical calculations resulting in NaN (not a number, printed as “nan” in Python) values. This value may also be used to indicate unavailable data. Computations to analyze such data may (but are not guaranteed to) propagate the NaN value to the final result where it is easy to spot. In particular, comparisons (<, >, =, ≠) involving NaN always evaluate to false, so operations that involve sorting data (e.g., computing minimum, maximum, or median) may fail and may silently produce incorrect results. An example is given in Table 1, produced by the code provided as Supporting Information S1 Data.

**Table 1:** Artifacts in data analysis using floating-point numbers. Using the Python script and data provided in Supporting Information S1 Data, we loaded the input data *x* and *y* in the top two rows (where one unavailable data point is interpreted as nan, floating-point "not a number"). Computing the ratio *x*/*y*, division by zero resulted in two further nonfinite values. Sorting the data (in ascending order, using Python's standard sorting algorithm) silently yielded an incorrect result. Computing the maximum based on successive comparisons failed as well, but this is not immediately obvious just from the value. Only the mean value is an obvious indication that something went wrong.

**Input data**										
	*x*	0.0	8.0	0.0	8.0	4.0	1.0	4.0	0.4	4.5
	y	1.0	1.6	0.0	2.0	0.0	—	1.6	0.4	1.5
**Analysis results (intermediate and final)**										
	Values *x*/*y*	0.0	5.0	nan	4.0	inf	nan	2.5	1.0	3.0
	Sorted values	0.0	1.0	3.0	5.0	nan	4.0	inf	nan	2.5
	Maximum	3.0								
	Mean	NaN								

### Incorrect data interpretation by computer programs

The two previous pitfalls can be seen as instances of the problem that computer programs interpret data incorrectly. Another pitfall is spreadsheet software inadvertently interpreting input data as dates (possibly in a foreign language date format) or floating-point numbers in scientific notation. For example [7], the supporting information of publications containing gene names or identifiers in spreadsheets (e.g., Excel) is prone to errors like "SEPT2" (Septin 2) becoming "2-Sep" or "2006/09/02.” This and the two pitfalls above are cases of inappropriate data processed by functions that are correct and useful for appropriate input. In addition, there is always the possibility of incorrect function, i.e., bugs, in any software [17].

This type of problem can sometimes be detected by plausibility checking. To avoid these types of errors, using different functions or different software can help, or you may need to clean up the input data. In case of seemingly plausible but actually incorrect results (e.g., the maximum reported in Table 1), only a very detailed look at the data can help reveal the problem. In this case, one should be glad about every error message or program crash.

## Correctly interpret your data

### Tip 4: Treat units with respect

Unless numbers are clearly dimensionless (e.g., relations between the same type of measurements, counts), they are meaningless without knowing the unit. For instance, what does a temperature "in the 30s" mean? A warm summer day at 30°C in Paris and you have a problem if the fridge in your lab is broken? Should you expect snow at 30°F in Seattle, and you could just store your specimens (or the lunch you brought) outside? Are you experimenting with superconductors at 30 K in a physics lab and need protective gloves? Besides single numbers, equations also only become meaningful if their units are clear: "A [mathematical] model without units is not a model" (Matthias König).

#### SI units in computations

Inconsistent units in computations are a typical source of error, in particular, if units from different sources are combined. Such errors can easily be spotted and corrected if they are merely a factor of 1,000, which typically leads to largely implausible results (e.g., using kilograms instead of grams). Implausibility can still be noted if the factor is different (e.g., 60 when using minutes instead of seconds), but the error is typically harder to find and correct in this case. Smaller errors bear the danger of going unnoticed, which can be the cause of serious problems: using pound seconds instead of Newton seconds as the unit of impulse led to the loss of the Mars Climate Orbiter probe [18], failing a multimillion dollar space mission.

An easy way to obtain consistent units is to use SI units everywhere in your calculation. Non-SI units should then be converted before starting the actual computations, and results can possibly be converted to intuitively understandable units at the end.

#### Intuitive units in presentation

For plausibility checking and presenting results, SI units with a suitable prefix may be intuitively understandable (like 7 μm instead of 0.000007 m when describing the diameter of red blood cells). In other cases, non-SI units might be more useful. For instance, a fuel consumption of 8 liters per 100 kilometers (or fuel efficiency of 29.4 miles per gallon) is easy to understand and seems plausible for a standard car. Converting this to SI units results in a fuel consumption of 8 × 10^−8^ m^2^, which is hard to interpret. Similarly, human water consumption of 2.6 liters per day is intuitively plausible. In contrast, 3 × 10^−8^ m^3^/s is hard to grasp and even confusing because it suggests continuous water uptake rather than drinking at irregular intervals.

#### Unit prefixes

Not only can units be a source of confusion but also SI prefixes of units and very large or small numbers. For instance, mistaking nano for 10^−12^ is wrong by three orders of magnitude, so this can possibly be detected by plausibility checking. However, plausibility checking is challenging for very small and very large numbers because they are hard to understand intuitively. For instance, it is not immediately obvious that one microcentury (about 53 minutes) is a useful upper limit for the length of presentations. The difficulty of intuitively grasping large or small numbers also applies to expressions such as parts per billion (ppb; 1 in 10^9^), which has the additional danger of confusion due to "billion" (or similarly spelled) meaning 10^12^ in some languages, e.g., French, German, and Spanish. Furthermore, confusion should be avoided between how units are used colloquially and scientifically. For instance, the trail mix that of one of the authors (LOS) was eating while writing this sentence has 523 kcal per 100 g, which would be grossly underestimated by saying "it has 523 calories."

#### Units in formulas

Considering the units used in formulas can help in two ways: when checking your own formulas and when trying to understand other people’s formulas.

When checking plausibility of your own formulas, one aspect is to check whether all units match. Incorrect units can flag forgotten constants and indicate what unit or constant would be needed so that units match. Similarly, when discussing a certain quantity, agreeing on the unit of said quantity is one way of making sure that everyone has the same understanding of what is being discussed. Quantities of different units cannot be added or subtracted (how much should two seconds plus half a meter be?). Multiplication and division of units is perfectly valid, provided that the overall unit of the formula makes sense. Differentiation, i.e., computing derivatives, corresponds to the division by the respective unit (typically time or length), e.g., velocity in the units meters per second is the temporal gradient (one over seconds) of position (meters). Conversely, integration corresponds to multiplication by the respective unit.

When dealing with formulas from literature, tracking units throughout the formula can help you understand the meaning of the formula and its constants. As an example, suppose you are studying the influence of cytochrome P450 enzymes on the metabolism of theobromine (e.g., [19])—the principle alkaloid in cacao. In the context of such enzyme kinetics, concentrations typically change according to Michaelis–Menten kinetics [20], a nonlinear change of a concentration *c* according to

$$\frac{dc(t)}{dt}=\dot{c}(t)=-\frac{V\cdot c(t)}{K_{m}+c(t)}.$$ (1)

(Readers familiar with Michaelis–Menten relations might know *V* denoted as *V*_max_. This is not consistent throughout the literature, we follow the standard from [21] here.) The left-hand side of Eq 1 consists of the time derivative (one over seconds) of a concentration *c* (concentration units, i.e., either mass or molar amount per volume). On the right-hand side, addition in the denominator implies that *K*_*m*_ is a constant also in units of concentration. As there is a factor *c* (concentration) in the numerator, *V* needs to be expressed in units of concentration per time, i.e., *V* is a rate of concentration change.

#### Separating unit conversions from functional relationships

It is useful to separate unit conversion (if needed) from the subsequent computation to avoid obscuring the the actual logic behind formulas and constants. Assume you would like to express theobromine concentrations from the example above in moles per liter and use the time unit seconds for the rates. If you are lucky, constants $\tilde{V}$ and ${\tilde{K}}_{m}$ are given as mass change per minute in Eq 1; if you are less lucky, you will find constants for other compounds in "international units," a frequent source of frustration for one of the authors (SR). As the average molar mass of theobromine is 180.164 g/mol [22, 23], you would end up with

$$\dot{c}(t)=-\frac{\frac{1\min\cdot 1\mathrm{mol}\cdot \tilde{V}}{60s\cdot 180.164g}\cdot c(t)}{\frac{1\min\cdot 1\mathrm{mol}\cdot {\tilde{K}}_{m}}{60s\cdot 180.164g}+c(t)},$$ (2)

which is clearly unnecessarily hard to read and understand.

### Tip 5: Verify your formulas

Relations derived from numerical data (e.g., descriptive models) are typically written as formulas. Formulas by other authors found in the literature may be more or less cryptic at first glance and need to be understood correctly before making use of them. Besides tracing units in formulas (cf. Tip 4), further techniques can be employed to check formulas for plausibility or to interpret them correctly. This involves both the dependency on variables and constants in the formulas.

#### Asymptotic behavior

It is useful to check the limits of the mathematical expressions for extreme or critical values, i.e., to check what results are obtained for very large or very small values (positive and negative infinity, if applicable), for zero, or for differences being zero. If only finite ranges of values make sense (e.g., a percentage between 0% and 100%), the boundaries of these ranges should be considered. In particular, it is worth checking whether it is plausible that some limit is reached asymptotically (e.g., trees growing until they reach a maximal size) or whether a periodic pattern is expected (e.g., circadian foraging rhythms of animals).

#### Individual constants and variables

As an example, let us again consider the Michaelis–Menten kinetics from Eq 1, with time dependency omitted here for simplicity,

$$\dot{c}=-\frac{V\cdot c}{K_{m}+c},$$ (3)

where the constants *V* and *K*_*m*_ are positive. Clearly, only concentrations between zero and some maximal concentration (maximum soluble amount or pure substance only) make sense, so we only need to check this range. For positive *c*, the right-hand side is negative. For *c* = 0, the numerator is zero (with finite denominator), so the overall rate is zero. These two observations make perfect sense if Eq 3 describes a decay: concentration decreases if some substance is present to start with; otherwise, nothing happens. The larger the constant *V*, the larger the decay if all other values stay the same. More precisely, the decay rate is proportional to this constant. For large *c*, we can divide both denominator and numerator by *c* and obtain *V* as the hypothetical limit. The other constant, *K*_*m*_, is a bit more difficult to interpret: *c* = *K*_*m*_ is the concentration at which half the maximal rate, Vc(c+c)=V2, is attained.

#### Scaling

Besides considering asymptotic behavior of formulas, it is also useful to think about the asymptotic scaling of entire mathematical expressions when changing a single variable. For instance, suppose you want to study signaling pathways leading to proliferation of cancer cells; more precisely, you are interested in interactions between pairs of inhibitors. At your institution, this involves a complicated bureaucratic documentation of the purchases in addition to the actual experimental work. There is one purchase report per inhibitor (linear scaling), whereas the number of combinations of two inhibitors (i.e., the number of individual experiments) scales quadratically with the number of inhibitors, *n*(*n* − 1)/2 = (*n*^2^ − *n*)/2 if *n* is the number of inhibitors. The latter will eventually dominate the overall effort for larger numbers of inhibitors.

#### Monotonicity

Monotonicity (i.e., consistently decreasing or increasing function output for decreasing or increasing input) should also be considered if the data analysis or interpretation involves the definition of custom quantities measuring the extent of certain effects. For example, suppose you want to quantitatively assess soil contamination based on the presence of metallophytes—plants that tolerate high levels of specific heavy metals [24]. Such a measure should not be termed "soil quality measure" but rather "soil pollution measure" because a larger presence of metallophytes indicates larger pollution.

### Tip 6: Know thy statistical methods

Descriptive statistics and statistical tests are frequently used when reporting numerical data and also represent a frequent cause of confusion. It is important to check the requirements for a given method or test, e.g., whether data are normally distributed when performing parametric tests that require normality. This already applies when reporting means and standard deviations, which has the implicit assumption that data are normally distributed. Otherwise, medians and inter-quartile ranges (or, graphically, box plots) might be more useful descriptions of the ranges of typical values. In this tip, we describe four further frequent causes of confusion when using statistical methods.

#### Reversed effects in data subsets

Even though percentages are rather simple descriptive statistical quantities, they may lead to confusion when evaluated on subsets of data. This effect is known as Simpson's paradox; see, e.g., [25] for some real-life examples. A simplified hypothetical example is given in Table 2: two treatments (A and B, e.g., two different drugs) were applied in two groups of patients (groups 1 and 2, e.g., adolescent and adult patients). Treatment A was consistently more successful in both groups viewed separately. However, evaluated for the combined group of patients, treatment B appears to be more successful. This paradoxical situation indicates that someone could have selected subgroups just to make treatment A look better. More likely, there is an additional confounding variable behind the data as well as some mechanism that should be understood to explain why it makes sense to consider the two groups separately. We refer to [26] for a more detailed discussion of how to deal with Simpson's paradox.

**Table 2:** Simpson's paradox (synthetic data). Two treatments (A and B) were applied in two groups (1 and 2) of patients. Treatment A seems to be more successful in each of the groups viewed separately (100 > 87.5 and 66.7 > 50). However, evaluated for the combined group of patients, treatment B appears to be more successful (75 < 80).

		Treatment A			Treatment B	
	Success	Total	Percentage	Success	Total	Percentage
**Group 1**	15	15	100%	105	120	87.5%
**Group 2**	30	45	66.7%	15	30	50%
**Combined**	45	60	75%	120	150	80%

#### Correlation and concordance

One typical use for statistical methods is analyzing whether two different data sets *x*_*i*_ and ${\tilde{x}}_{i}$ are correlated, e.g., different quantities measured in related observations or the comparison of a new or simpler measurement technique compared to an accepted gold standard. There are different forms of relation between *x*_*i*_ and ${\tilde{x}}_{i}$ one may be interested in, from a generic relationship to the question of whether two measurement techniques yield the same results. Typically, a quantification by a correlation coefficient (CC) is desired.

The standard and frequently used Pearson CC [27] is applicable only in some cases because it measures whether there is an affine-linear relationship between the two data sets (i.e., whether ${\tilde{x}}_{i}$ differs from *x*_*i*_ by scaling *a* and an offset *b*, ${\tilde{x}}_{i}=ax_{i}+b$, where *a* transforms units of *x* to $\tilde{x}$ and *b* is in units of $\tilde{x}$) but nothing more or less. This relation could, e.g., be observed when measuring temperature in degrees Celsius and Fahrenheit. On the one hand, Pearson CC cannot capture more general relationships. If the relationship is not monotonic (e.g., periodic), it needs to be guessed or known to allow a comparison; e.g., volume should be proportional to diameter cubed. Correlation for general monotonic relationships can be quantified by the Spearman CC [28], e.g., if concentration and its change rate in enzyme kinetics according to Eq 1 were measured somehow. On the other hand, when checking *x*_*i*_ and ${\tilde{x}}_{i}$ for equivalence (e.g., when comparing measurement techniques), Pearson CC is too generic. Equivalence requires scaling by *a* = 1 and an offset of *b* = 0. For quantifying correlation in this case, the concordance CC [29, 30] is more suitable.

**Fig 2:**
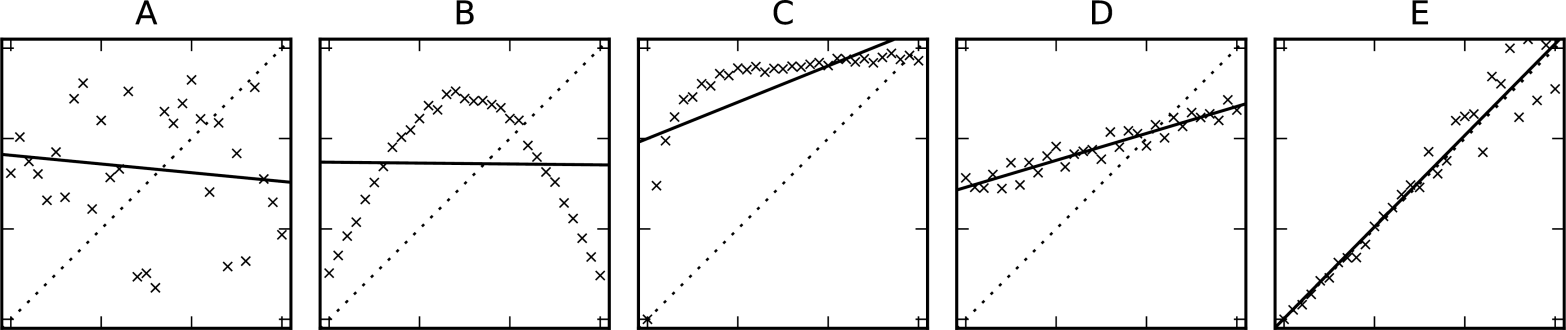
Data sets with different type of correlation. The data points in the five plots show different types of correlation between values on the horizontal and vertical axes: no correlation at all (panel A), a slightly noisy non-monotonic relationship (panel B), a nonlinear monotonic relationship (panel C), a linear relationship (panel D), and high agreement (panel E). The corresponding Spearman, Pearson, and concordance CCs are shown in Table 3. The solid line indicates the affine-linear fit; the dotted line is the identity (*y* = *x*).

Examples of generic, linear, and identical dependence between three different data series are shown in Fig 2, along with the corresponding CCs (Table 3). The code for this example is provided as Supporting Information S2 Data.

**Table 3:** CCs for the data sets shown in Fig 2. Uncorrelated data (A) and slightly noisy data following a clear nonmonotonic relationship (B) show poor CCs in all cases. A nonlinear but monotonic relationship (C) is captured by the Spearman CC but yields low Pearson CC. A linear relationship is characterized by high Pearson CC (D, E), but only a good agreement between the two data series (E) yields a high concordance CC.

Data Series	Spearman CC	Pearson CC	Concordance CC
A: Pure noise	0.108	0.128	0.120
B: Nonmonotonic	0.019	0.013	0.012
C: Monotonic, nonlinear	0.955*	0.639	0.280
D: Linear	0.951	0.948*	0.459
E: Identical	0.971	0.972	0.971*

*Indicates the suitable CCs for cases E to E, respectively.

**Abbreviation:** CC, correlation coefficient.

#### Correlation does not imply causation

If data series measuring two different effects are found to be correlated, this should be interpreted with care. Correlation may mean that one effect most likely causes the other (e.g., the frequency of smoking and the risk of lung cancer) or that there is an underlying cause for both effects (e.g., increasing sales of ice cream and increasing number of swimming accidents, both due to the summer holiday season). Correlated effects, however, may also be entirely unrelated (such as the decreasing number of pirates and global warming [31, p. 34], or the number of people drowning after falling into a pool and the number of films Nicolas Cage appeared in per year [32]).

#### Misconceptions about statistical significance

Statistical significance, usually quantified by *p*-value, should not be over-interpreted. It is a topic of ongoing debate to what extent statistical testing is being abused (see [33] and references 21–74 therein) and to a dispute to what extent false findings based on statistical testing have been published [34–36]. This has led some scientific journals to discourage reporting statistical significance [33].

Statistical significance at a given level in typical applications means that two series of measurements are likely to be different (i.e., the null hypothesis "they are the same" can be rejected with a certain level of confidence). The typically chosen threshold, *p* < 0.05, is arbitrary [37]; it is only used commonly because it is used commonly. It is important to keep in mind that

- A small *p*-value does not prove that there is a meaningful difference;
- A small *p*-value ("statistically significant") does not imply that the difference is big or relevant ("biologically significant");
- A smaller *p*-value does not imply a bigger difference; and
- A large *p*-value does not prove the absence of a relevant difference.

We refer to [33] for further misconceptions about *p*-values.

#### Data dredging

Moreover, "achieving significant findings is merely a question of looking hard enough" [38]. The authors of [38] illustrate this by an example that being born under the sign of Pisces significantly increased the 90-day survival among patients with severe sepsis and in need of fluid resuscitation. This approach is called "*p*-value hacking" or "*p*-hacking" (see, e.g., [39]), and constitutes one way of "data dredging" (see, e.g., [40]). As an additional example, we investigated baseball players born in Canada and the United States as described in Supporting Information S1 Text, using the Lahman Baseball Database [41] (subject to the CC-BY-SA license [42]). We found that players born in June were more often "[c]aught [s]tealing" than those born in September (median 9.0 versus 5.0, 277 versus 348 players, *p* = 1.32 × 10^−4^) and that players who eventually died on day 9 of any given month more frequently "[s]acrifice[d] hits" than players who eventually died on day 27 (median 19.5 versus 6, 168 versus 168 players, *p* = 3.02 × 10^−5^). Data and code to obtain these results are provided as Supporting Information S3 Data. To emphasize that anyone can obtain such highly significant results, we point out that the author performing this analysis (LOS) did not know what "being caught stealing" or "sacrificing a hit" means and made no attempt to find out at the time of writing.

### Tip 7: Keep track of accuracy

Besides tracking where values come from (cf. Tip 2), it is also important to keep track of how accurate they actually are. (Keep in mind that 68.432% of all statistics pretend to be more accurate than they actually are.)

#### Sources of errors and propagation of uncertainty

Errors need to be tracked throughout the entire data acquisition and analysis workflow. Measurements are typically subject to measurement errors whose extent strongly depends on the measurement technique employed, leading to uncertainty in the measured values [43]. The main causes for errors and uncertainty are systematic errors (e.g., miscalibrated measurement devices), limited resolution of measurements, and random errors. Moreover, the typically limited number of measurements taken leads to a sampling error that should be taken into account when generalizing findings. For instance, measuring blood pressure a few times a day provides important information for patients with hypertension but provides neither the time course for an entire day nor a detailed pressure curve during a single heart beat. Similarly, the images in Fig 4 below only show a limited spatial sample for the flower density in the entire respective meadows.

For simplicity, we will consider only uncertainty due to random errors assumed to be normally distributed. Relative uncertainty of a single measurement (as a percentage) can then be expressed as the coefficient of variation, i.e., standard deviation divided by mean value. Uncertainties propagate through calculations [44], which may render results meaningless in the worst case. For instance, it is a hopeless endeavor to try to quantify differences in the range of 3% in a small sample of data with uncorrelated measurement uncertainty in the range of 20%.

As an example of uncertainty propagation, suppose you are trying to estimate the (ground) speed of a flock of flying white storks (*Ciconia ciconia*) without sophisticated equipment. Time measurement using your smartphone is probably possible with an uncertainty of less than a second. In contrast, even though global positioning system (GPS) helps you measure distances on the ground, estimating the distance of the birds in the air is much more difficult. Assuming you measured a flight path of one kilometer with an uncertainty of 10% and time of 100 s (i.e., 1% accuracy), the resulting speed (distance divided by time) of 10 m/s has an uncertainty of $\sqrt{10^{2}+1^{2}}=10.05\%$ [45] (assuming small errors with normal distribution and no correlation between the measurement uncertainties). In this example, the error in the distance measurement dominates the overall error. (Following Tip 1, you will clearly notice that this example uses simplified and artificially round numbers. However, 10 m/s, or 36 km/h, intuitively seems in a realistic range and agrees with literature values [46].)

#### Uncertainty quantification

Depending on the complexity of formulas in the data analysis, the uncertainty in the final result (or sensitivity of the final result against noise in the input data) may be hard to investigate analytically. In this case, Monte Carlo simulations for quantifying uncertainty (see, e.g., [47]) may be a useful technique: First, estimate the extent of uncertainty (e.g., measurement errors) to be expected for the raw data. If you have no better idea about the distribution of errors, assume normally or uniformly distributed noise at this point. Using this estimate, add random noise to the actual measurements to obtain synthetic input data for the data analysis. Then, analyze different sets of such synthetic data and quantify the variation obtained in the final result. In Supporting Information S4 Data, we implemented a Monte Carlo simulation in Python to estimate the uncertainty in the stork flying speed example, confirming the analytic result above.

This uncertainty quantification (UQ) serves two purposes. On the one hand, UQ indicates to what extent conclusions can or should be drawn from the results. On the other hand, UQ gives an idea how many digits of numerical results are significant and should be presented in text and tables.

### Tip 8: Don't rely on numbers alone

**Fig 3:**
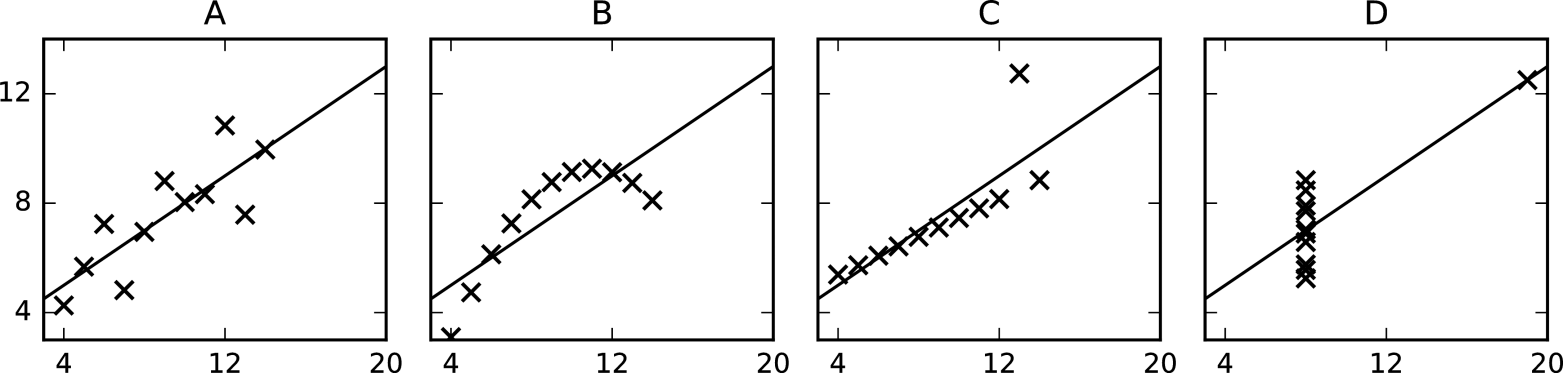
Anscombe's quartet (plotted). Anscombe’s quartet [48] is a classical example of four visually qualitatively different data series that become almost indistinguishable when computing simple summary statistics (see Table 4).

Even when properly considering uncertainty in numbers (cf. Tip 7), quantitative descriptions of data should not be the only thing you look at. Clearly, it is typically necessary to simplify raw data for a useful presentation and to reach useful findings, but simplification may also lose relevant information and conceal parts of the bigger picture. For instance, Fig 3 shows a classical example of four clearly different data series which are indistinguishable based on common measures from descriptive statistics (mean and standard deviation in both dimensions) (see Table 4). The code for generating these plots is provided as Supporting Information S5 Data.

**Table 4:** Anscombe's quartet (summary statistics). The data series x, y (plotted on the horizontal and vertical axis, respectively, in Fig 3) become almost indistinguishable when computing simple summary statistics.

	Series A	Series B	Series C	Series D
Arithmetic mean $\bar{x}$	19.0	19.0	19.0	19.0
Variance $s_{x}^{2}$	11.0	11.0	11.0	11.0
Arithmetic mean $\bar{y}$	17.5	17.5	17.5	17.5
Variance $s_{y}^{2}$	14.127	14.128	14.122	14.123
Pearson CC *ρ*_*x*,*y*_	10.816	10.816	10.816	10.817

**Abbreviation:** CC, correlation coefficient.

#### Don't miss the full picture

**Fig 4:**
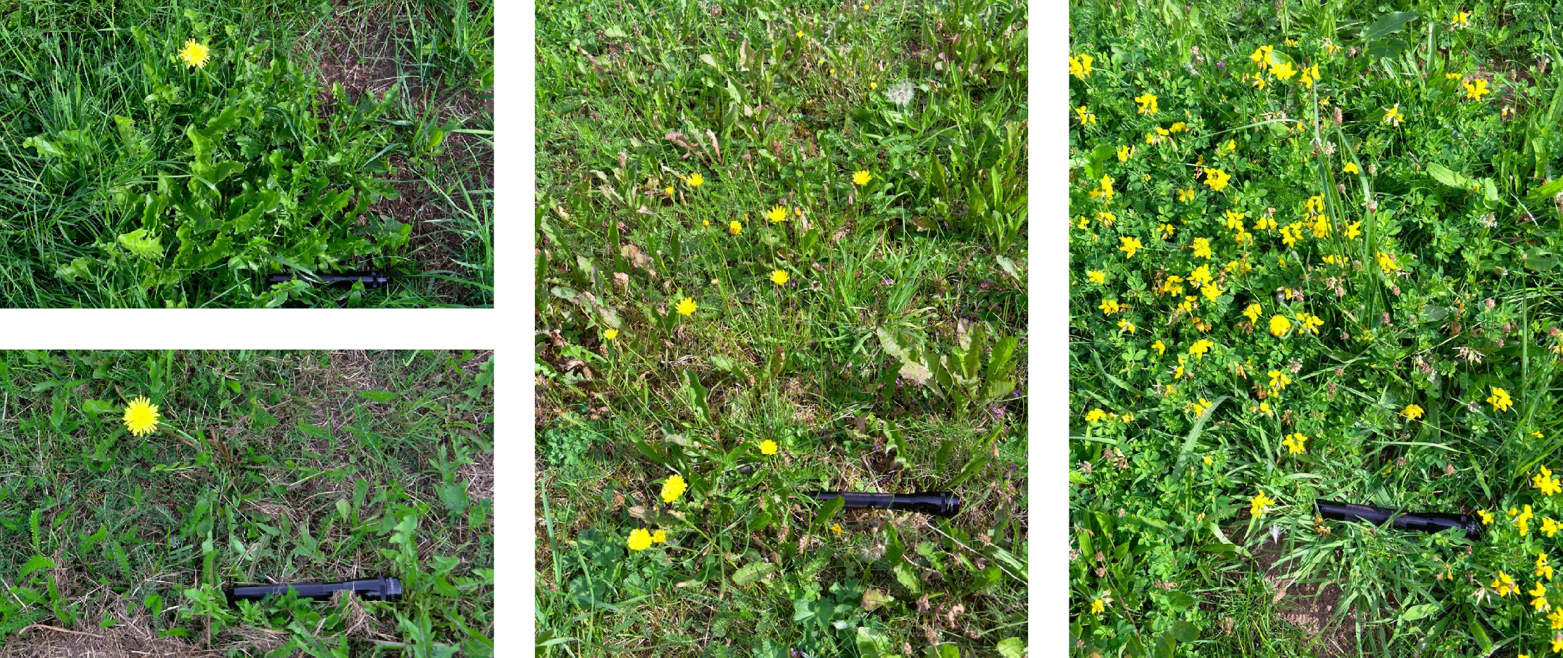
Counting flowers gone wrong. Consider a simple algorithm counting the number of dandelions in digital images based on the number of yellow pixels in the image and the scale indicated by the black rod (actually a flashlight). After calibration, using the two images on the left, the algorithm happily reports that there are approximately five and ten dandelions in the middle and right image, respectively. The fact that the yellow patterns actually show different species of plants could relatively easily be detected by visual inspection of the data. Algorithmically distinguishing the plants could also be possible but would take substantially more effort than we spent here on simple pixel counting. The code and the original images are provided as S6 Data.

At least for an internal assessment, one should always try to look at as much of the data as possible to decide what part of the contained information is possibly relevant and where simplifications are feasible. Spotting relevant patterns in graphical representations of data is typically much easier than in numbers alone, so suitable visualization is usually helpful before further computational assessment. For instance, in the assessment of histological specimens, patterns in the stained markers (e.g., proliferating cells) or the localization of such cells relative to other structures in the image provide much more information than a mere average cell density. A related botanic example is shown in Fig 4, in which applying an (unrealistically simplified) quantification approach to inappropriate input data yields incorrect results.

It depends strongly on the type of data which type of visualization is suitable for an internal assessment. To get an overview of bivariate data points, a simple scatter plot may be sufficient. To view time series, a line plot with interactive zooming to relevant ranges can be useful. Data with spatial dependence should be visualized in a way reflecting this spatial property; otherwise, patterns might be lost. These visualizations do not yet need to be of publication quality; they mainly need to be useful for the author (but should, of course, be improved later if they turn out to be useful).

Based on a visualization and other considerations, one can choose useful groupings and simplifications of data to look at. For instance, in a study on drug efficacy based on specific parameters measured in the blood before and after treatment, one could simply compare the averages over the entire cohort ("20% faster recovery!"). This may, however, be misleading if the response (comparison of the parameters before and after treatment) varies individually ("50% of the patients recovered twice as fast, the other half took even longer").

#### Don't overinterpret numbers

Single numerical values cannot accurately represent multiple criteria at the same time, and the objectivity of numbers in general should not be over-interpreted. Human body mass index (BMI), e.g., measures precisely one thing (body mass divided by the square of size) but does not distinguish between bone, muscle, and fat portions of the body mass. Even if BMI correlates more or less strongly with other health indicators, they are by no means a meaningful surrogate of an overall "health measure." The commonly used impact factor (IF) of scientific journals seems highly objective at first glance but is also prone to over-interpretation. The IF is not an immediate measure of "quality" (whatever a precise definition would be), values of IFs largely differ between disciplines, and the standard IF is easily confused with measures defined for similar purposes—serious ones [49] as well as dubious ones used in advertisements by fishy journals (see your local spam folder).

#### Only calculate where feasible

Values of numerical measures following on nonproportional and ordinal scales should be compared with care: just because something is a number, it is not necessarily usable for use in calculations. For instance, a temperature of −20°C is not "twice as cold" as −10°C, and a CC of *ρ*_1_ = 0.9 is not "fifty percent better" than *ρ*_2_ = 0.6. Similarly unsuitable for calculations are medical scores, summarizing and simplifying diagnostic observations, e.g., to an integer between 1 and 6, to support therapy decisions. For example, a Gleason score for prostate carcinoma (see, e.g., the study [50] with a rather fitting title) of 4 versus a score of 2 cannot be interpreted as "double size" or "twice as dangerous."

Besides distinguishing numerical from ordinal quantities, it is also useful to distinguish extensive and intensive quantities [51]. An extensive quantity of an object is split if the object is split, e.g., mass or volume. An intensive quantity remains the same if an object is split, e.g., temperature or concentration. When combining objects, extensive quantities are added. As an example, combining 2 liters of water at 30°C and 1 liter of water at 60°C results in approximately 3 liters (extensive) of water at 40°C (intensive). In this example, a weighted average of the intensive quantities is obtained. This is not generally true for intensive, e.g., when combining objects of different melting points. Note, moreover, that this result for water is only approximately correct due to nonlinear thermal expansion and contraction.

## Present your data in a useful way

### Tip 9: Properly present data

To properly present results based on numerical data, two aspects need to be considered: what are the best numbers to present for supporting your findings? What is the best way to present numbers?

#### Values, absolute differences, and relative differences

Many studies investigate differences in one way or another. Depending on the application, absolute values, absolute differences, or relative differences can be the best way to present results. For instance, human body temperature is typically reported as, e.g., 37.6°C (absolute value) rather than as a difference to "normal" body temperature; an overweight person (125 kg) will probably report losing 10 kg (absolute difference) rather than 8%; the success of an agricultural wheat cultivation technique is best understood by saying that it yielded, e.g., 20% more crops than prior techniques. To understand what any difference means, "normal" values (the baseline) should be stated as well. In the examples above, normal human body temperature (around 37°C) and a range for normal body weight (depending on size, e.g., 75 kg) is probably known to most readers, whereas few people are likely to know the typical amount of wheat harvested per area—about 6 metric tons per hectare (value for Austria in 2014 [52]).

#### Numbers, tables, and plots

Depending on the amount of data you need to present to sustain your findings, a suitable form of presentation should be chosen. Single numbers can just be written in the text; few numbers whose exact values are needed can be put in a table; time courses or larger sets of data are typically best presented in plots. It can be useful to retain spatial information when graphically presenting spatial data. For instance, showing infection rates per country in a colored map might make comparison of values more difficult but can indicate geographical patterns. It can be useful to limit the amount of detail for data presented in an article when providing more detailed numbers and raw data as supplementary information; cf. Tip 10.

**Fig 5:**
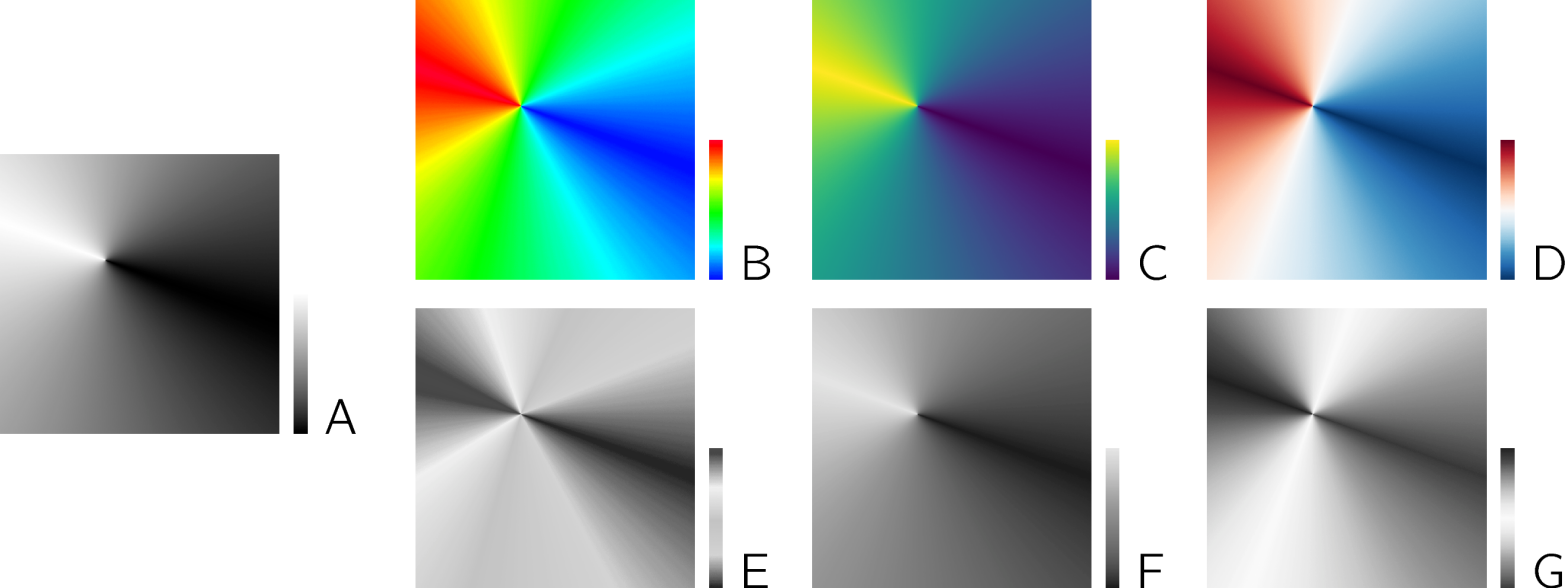
Different color maps used for visualizing the same pseudo-data. (A) The sequential "rainbow" color map (panel B) is frequently used and thus easy to interpret but poorly conveys absolute values (cf. the "rays" in yellow and cyan) and does not preserve information well if printed in gray scale (simulated by conversion to gray scale in panel E). The sequential "viridis" color map (panel C) is a perceptually uniform color map avoiding such artifacts and translating better to gray scale (panel F). The divergent color map from blue via white to red (panel D) is useful if the direction and the extent of deviation from a central value is of interest and loses only the direction information when converted to gray scale (panel G).

For standard plots, logarithmic rather than standard linear axes can be useful, in particular, if ratios between values are of interest. In this case, linear axes may be misleading because parallel lines (indicating the same absolute change, e.g., from 0.5 to 1 and from 3.5 to 4) can be misinterpreted as the same relative change. When using color maps to present numerical data, two aspects should be considered. If a central value of the data range has a specific meaning (e.g., "unchanged expression" when visualizing up-regulation or down-regulation of genes from microarray data), a diverging color map should be used rather than a standard sequential one. Moreover, color maps should be chosen such that the relevant information is still conveyed if the figure is printed in gray scale or viewed by a person with impaired color vision. An example for the effects of different color maps is shown in Fig 5. We refer to [53, 54] for further details on the choice of suitable color maps.

**Fig 6:**
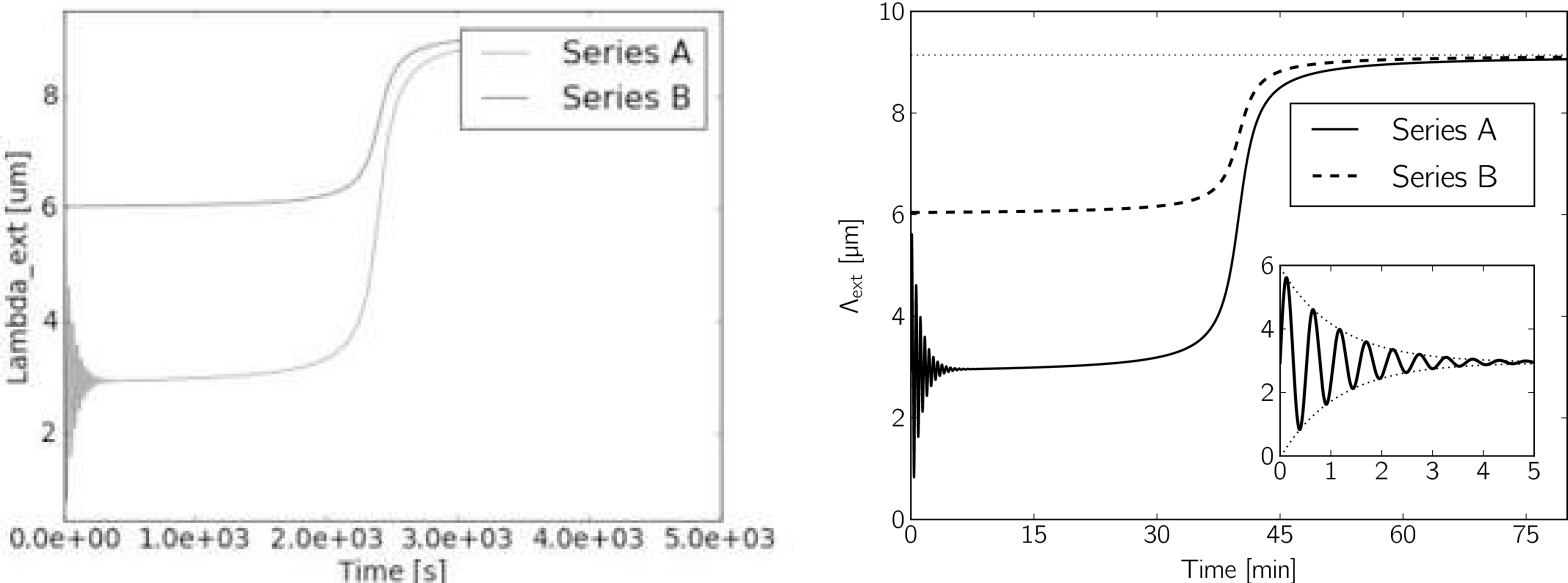
The same two synthetic time series Λ_ext_ plotted in two different ways. Compared to the right plot, the left plot is of rather poor design and quality. The text in the plot is inconsistent with the rest of the manuscript (symbols like Λ and μ), numbers are unnecessarily hard to read and not in intuitively understandable units, the two curves of originally different color are indistinguishable in this simulated printout on a black and white printer; the legend occludes part of the data, the possibly interesting oscillation is of the lower curve hard to see and raises more questions than it answers; the range of values starts at almost (but not quite) zero; the image is rather blurred and contains lots of artifacts due to low resolution, resampling, and (lossy) jpeg image compression. In contrast, the right plot is crisp and clear, additionally shows the asymptotic value attained by both data series, and shows a zoom to how the damped initial oscillation of data series A (here, we can actually tell which one is which).

Generally, the presentation of results—in particular plots—should be designed in a way that does not confuse the reader, avoids ambiguity, and shows a clear message but does not show too many things at once. Only poor-quality results deserve a poor-quality presentation, whereas correct and well-designed figures give the impression that you thought about and you understood what you did. Fig 6 shows an example of the same data presented in different quality. The code for generating these plots is provided as Supporting Information S7 Data. For more details on suitable ways of plotting data, we refer to [5].

### Tip 10: Share your data

Data analysis and presentation typically strongly reduces the amount of data that is finally reported (in a presentation, on a poster, in an article or thesis). Hence, it may make sense to also provide more of the original data (unless there are, e.g., legal restrictions or privacy concerns) and intermediate results to the interested reader, for example, as supporting information of publications, or via domain-specialized or generalist data repositories (see [55] for an overview; generalist repositories include Figshare [56], Harvard dataverse [57], the Open Science Framework [58], and Zenodo [59]). Providing data and code (e.g., scripts) used for data evaluation increases reproducibility and credibility of your results.

Publishing data via reliable platforms also ensures long-term availability of research results, which is a challenge otherwise [60]. Making research data, code, and results freely available ("open access" and/or "open data") is of increasing interest to funding agencies, e.g., the European Union [61]. Publishing research data is also encouraged to a different extent by many journals and publishers [62], including *PLOS* [63].

Preparing data and code for publication certainly requires some effort, but one also benefits from this work in different ways. Before being published, data and scripts need to be documented and prepared, e.g., in a suitable standardized format. This can help detect mistakes by simply looking at the material again but primarily helps make data and code understandable to other people (e.g., your immediate colleagues or even yourself a few months from now). Moreover, published data sets and scripts are also guaranteed to be available to yourself, e.g., after moving to a different lab. Using open source platforms for implementing data evaluation helps make such scripts reusable by yourself and others; cf. Tip 9 in reference [64]. Another aspect of sharing data and code is that other researchers might actually use it. This may help your work to get recognized and cited, fruitful new cooperations might evolve, and others might find gems in your data you will never even start looking for.

Following this recommendation, we provide the data and code used for our analyses in the supporting information to this article. Although we do not believe that there are further gems hidden in our data (the baseball data is not ours), we are looking forward to being proven wrong.

## Supporting information

S1 Text Description of the *p*-value hacking in Tip 6. (PDF)

S1 Data Data and code for the floating point number example in Table 1 in Tip 3. The results were obtained using Python 3.5.2, with Numpy 1.11.3 and Pandas 0.19.2.
(TGZ)

S2 Data Data and code for the correlation plots in Fig 3 in Tip 6. (TGZ)

S3 Data Data and code for *p*-value hacking in Tip 6 and Supporting Information S1 Text. This archive contains selected tables of the Lahman Baseball Database [41] (in comma-separated values format) by Sean Lahman, licensed under a Creative Commons Attribution-ShareAlike 3.0 Unported License [42], and our Python code implementing the calculations described in Supporting Information S1 Text.
(TGZ)

S4 Data Code for the Monte Carlo simulation for UQ in Tip 7. UQ, uncertainty quantification.
(TGZ)

S5 Data Code for the anscombe plots in Fig 4 in Tip 8. (TGZ)

S6 Data Data and code for the flower counting example in Tip 8. This archive contains geo-referenced images used for the flower counting example in Fig 2 and the Python script implementing the pixel counting described in the caption to Fig 2.
(TGZ)

S7 Data Code for plots in Fig 4 in Tip 9. (TGZ)
