# Supplementary material for: Ten quick tips for getting the most scientific value out of numerical data
Source: PLoS Comput Biol. 2018 Oct 11;14(10):e1006141. doi: 10.1371/journal.pcbi.1006141 (PMC6181270; doi:10.1371/journal.pcbi.1006141)
Supplement: S1 Text — (PDF) [file pcbi.1006141.s001.pdf]

Ten Quick Tips for
Getting The Most Scientific Value out of Numerical Data

Supporting Information S1 Text

Lars Ole Schwen   Sabrina Rueschenbaum

p-Value Hacking in Tip 6

To demonstrate that anyone can find statistically highly significant results, a domain non-expert (LOS) searched a database for Major League Baseball for significant differences between subgroups of players, abusing statistical techniques by p-value hacking.

Methods. For this purpose, we filtered the Lahman Baseball Database [41] to include only US- and Canadian-born players. Then, we grouped the players in five categories: day and month in the birth date of players, day and month in their death date, and state in which they were born, all subject to availability of the respective individual data. This resulted in $2 \times (12 \times 11) + 2 \times (31 \times 30) + 60 \times 59 = 5664$ subgroups of players. 54 numerical data columns (i.e., up to 54 numerical data values per player) describing different aspects of player performance were present in the database. The data contained unavailable values as well as zero values, whose validity we could not determine without further (undesired) understanding of the data, hence we dropped this data in the further analysis.

In each category, we retrieved data from each data column separately for each group and checked all distinct groups for significant differences. We had no reason to assume a specific distribution of the data, normal or otherwise, so we used the Mann–Whitney u test [28] to test for significant differences in a total of $5664 \times 54 = 305\,856$ cases. We finally sorted the results by p-value in each category. The three pairs of groups with smallest p-values per category are reported in Table 1.

Implementation. The Python code and data used for p-value hacking are provided as Supporting Information Data S3.

Assessment of the Results. It seems plausible that there should not be substantial differences between players who, e.g., eventually died in different months. However, we also cannot rule out that performance data correlates with the groups at least to some extent. For instance, players born in January are, on average, slightly older than those born in December, which might have an impact on some performance measures. Similarly, it is conceivable that

Description		Group A				Group B			approx. p-value
	group	n	Med	IQR	group	n	Med	IQR	
Birth Day (L^2dist $= 2.66 \times 10^{-3}$)									
b-IBB	day 11	83	7.0	18.0	day 27	88	16.5	29.25	1.60×10^{-4}
b-IBB	day 24	77	5.0	21.0	day 27	88	16.5	29.25	2.87×10^{-4}
b-IBB	day 23	75	6.0	16.0	day 27	88	16.5	29.25	2.92×10^{-4}
Birth Month (L^2dist $= 6.85 \times 10^{-3}$)									
b-CS	June	277	9.0	21.0	Sep.	348	5.0	14.0	1.32×10^{-4}
f-GS	June	471	76.0	349.0	Sep.	638	43.5	239.5	1.95×10^{-3}
b-IBB	Jan.	210	6.0	18.0	June	199	12.0	32.0	2.10×10^{-3}
Birth State (L^2dist $= 1.91 \times 10^{-3}$)									
b-G	CA	2182	121.5	404.5	PA	660	33.0	217.0	6.74×10^{-27}
b-G	CA	2182	121.5	404.5	MA	1410	52.0	276.0	1.54×10^{-25}
f-G	CA	2167	107.0	363.5	PA	648	33.0	203.5	2.92×10^{-23}
Death Day (L^2dist $= 2.92 \times 10^{-3}$)									
b-SH	day 9	168	19.5	39.0	day 27	168	6.0	20.25	3.02×10^{-5}
b-H	day 9	248	56.5	363.75	day 27	250	23.0	141.5	3.95×10^{-5}
p-BK	day 2	46	1.0	1.0	day 12	37	2.0	2.0	8.56×10^{-5}
Death Month (L^2dist $= 7.49 \times 10^{-3}$)									
f-GS	May	56	27.5	116.0	Oct.	62	113.0	191.25	2.49×10^{-3}
b-SH	April	408	8.0	21.0	Sep.	363	11.0	29.0	2.89×10^{-3}
b-SH	March	402	11.0	27.25	April	408	8.0	21.0	3.56×10^{-3}

Table 1: Results of p-value hacking for baseball statistics data. (Med: Median, IQR: interquartile range, L^2dist: L^2 distance between the cumulative distribution function (CDF) of p-values in category and a uniform CDF according to Eq (1), b-IBB: batting/intentional walks, b-CS: batting/caught stealing, f-GS: fielding/games started, b-G: batting/games, f-G: fielding/games, b-SH: batting/sacrifice hits, b-H: batting/hits, p-BK: pitching/balks, Jan.: January, Sep.: September, Oct.: October, CA: California, PA: Pennsylvania, MA: Massachusetts)

teams in certain states (which probably tend to have more players from the given state) prefer certain tactics, which might also lead to differences in the data.

To investigate whether the observed p-values are really random findings or indicate actual correlations in the data, we further analyzed the distribution of p-values obtained in each category. If the data considered in these comparisons is fully random, the p-values are uniformly distributed in $[0, 1]$ [65], so a certain number of small p-values can be expected without any actually relevant differences between the groups. However, if there is correlation in the data, there will be more significant differences than for random data and thus smaller p-values than for a uniform distribution. Hence, we computed the L^2 difference between the cumulative distribution function CDF_p of the p-values obtained and the identity (CDF of a uniform distribution). If $0 \leq p_1 \leq \cdots \leq p_N \leq 1$ are the p-values in non-descending order, this can be computed as

$$\sqrt{\int_0^1 \left(\mathrm{CDF}_p(x) - x\right)^2} \approx \frac{1}{N}\sqrt{\sum_{i=1}^{N} (p_i - i/N)^2}. \tag{1}$$

This difference is smaller than 0.008 for all five categories (cf. Table 1), indicating that our seemingly significant results are actually random findings. Only for the results sorted by birth state, surprisingly low p-values are obtained when comparing the number of games played by California-born players to those born in other states. We will refrain from speculation what this might mean.

Discussion. From a statistical point of view, going fishing for significant results by testing thousands of hypotheses is clearly a form of data dredging or p-value hacking, and thus scientifically absolutely unacceptable. Using non-parametric testing for the comparison of individual subsamples, however, is statistically sound and would be a suitable approach if we were testing single, well-founded hypotheses for the data at hand. Similarly, comparing the CDFs of the p-values to those of a uniform distribution seems to be suitable for verifying that our "highly significant results" are probably actually random findings, except for the results for the birth state category with very small p-value.

The least one should do when testing multiple hypotheses is using Bonferroni correction [66], dividing the basic significance level by the number of hypotheses tested. In our case, we need to consider the five categories from Table 1 separately, since they are of different size. This would translate the standard significance level of 0.05 to $0.05/(31 \cdot 30) \cdot 1/5 \approx 1.08 \times 10^{-5}$ for birth/death day (approximately, as not every month has 31 days), $0.05/(12 \cdot 11) \cdot 1/5 \approx 7.58 \times 10^{-5}$ for the birth/death month categories, and $0.05/(60 \cdot 59) \cdot 1/5 \approx 2.83 \times 10^{-6}$ for the birth state category. In this case, only the findings in the birth state category remain significant, confirming that the "highly significant results" are probably just random findings. Still, Bonferroni correction should not be used as an excuse to go fishing in the dark for significant results.

References

[28] Corder GW, Foreman DI. Non-Parametric Statistics for Non-Statisticians: A Step-By-Step Approach. 2nd ed. Wiley; 2014.

[41] Lahman S. The Lahman Baseball Database, 2014 Version; 2015. Licensed under a Creative Commons Attribution-ShareAlike 3.0 Unported License. Available from: https://github.com/chadwickbureau/baseballdatabank.git.

[65] Hung HMJ, O'Neill RT, Bauer P, Kohne K. The Behavior of the P-Value When the Alternative Hypothesis is True. Biometrics. 1997;53(1):11–22. doi:10.2307/2533093.

[66] Bland JM, Altman DG. Multiple significance tests: the Bonferroni method. BMJ. 1995;310(6973):170. doi:10.1136/bmj.310.6973.170.
