# Supplementary figures and images for: Ten quick tips for getting the most scientific value out of numerical data
Source: PLoS Comput Biol. 2018 Oct 11;14(10):e1006141. doi: 10.1371/journal.pcbi.1006141 (PMC6181270; doi:10.1371/journal.pcbi.1006141)

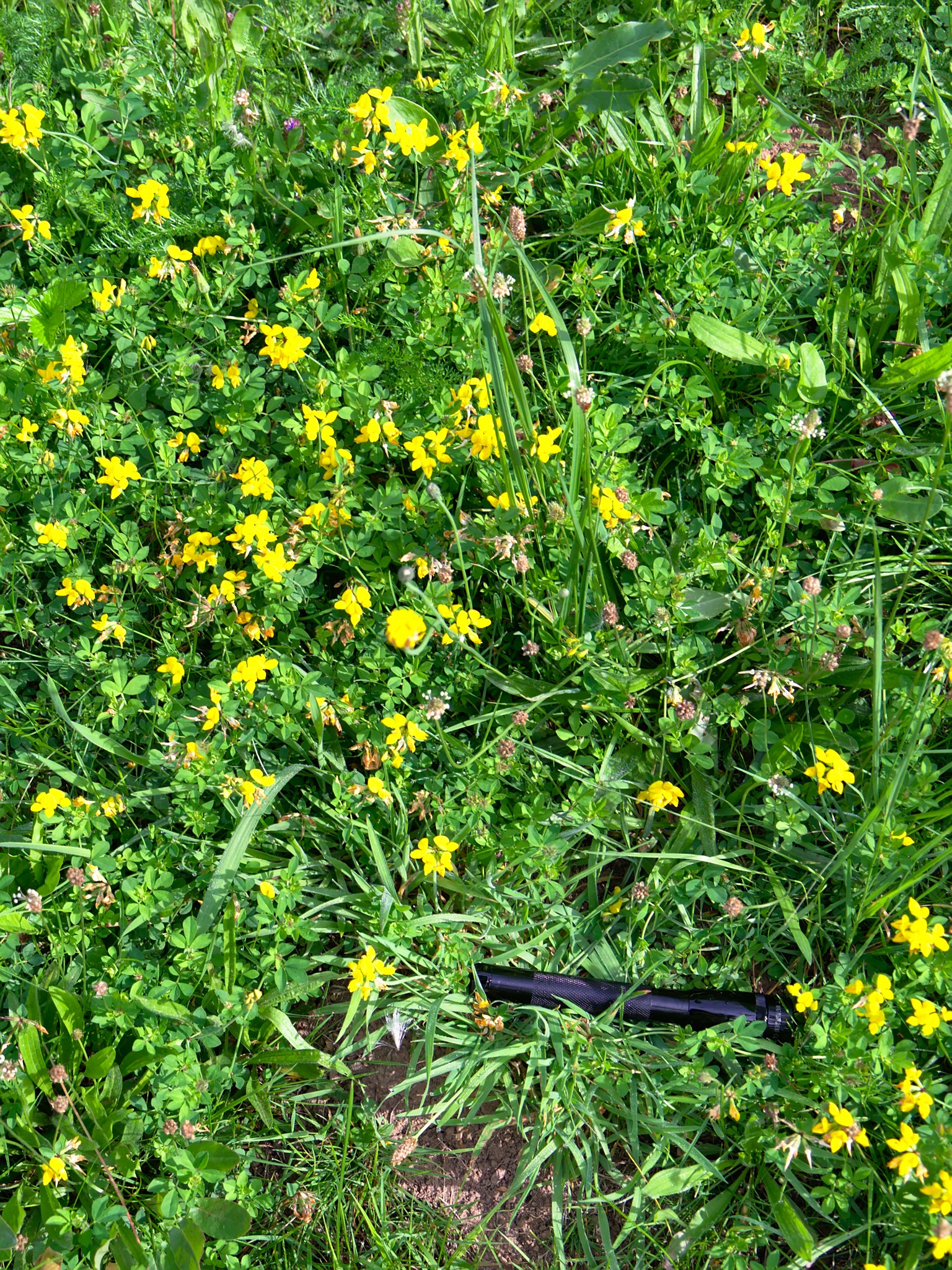

Supplement: S6 Data — This archive contains geo-referenced images used for the flower counting example in Fig 2 and the Python script implementing the pixel counting described in the caption to Fig 2. (TGZ) [file pcbi.1006141.s007.tgz › flowerExample/DSC_5883.jpg]

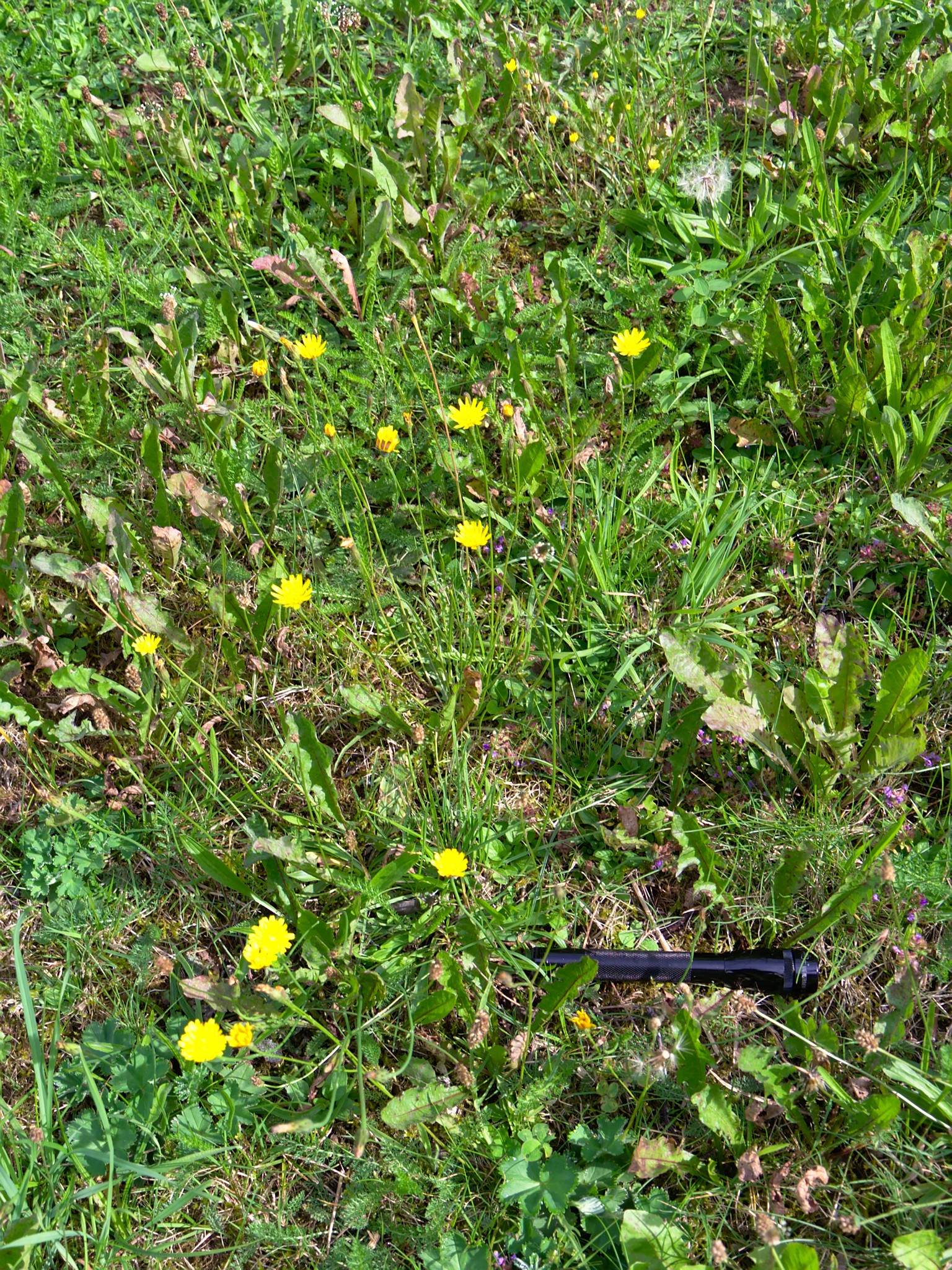

Supplement: S6 Data — This archive contains geo-referenced images used for the flower counting example in Fig 2 and the Python script implementing the pixel counting described in the caption to Fig 2. (TGZ) [file pcbi.1006141.s007.tgz › flowerExample/DSC_5884.jpg]

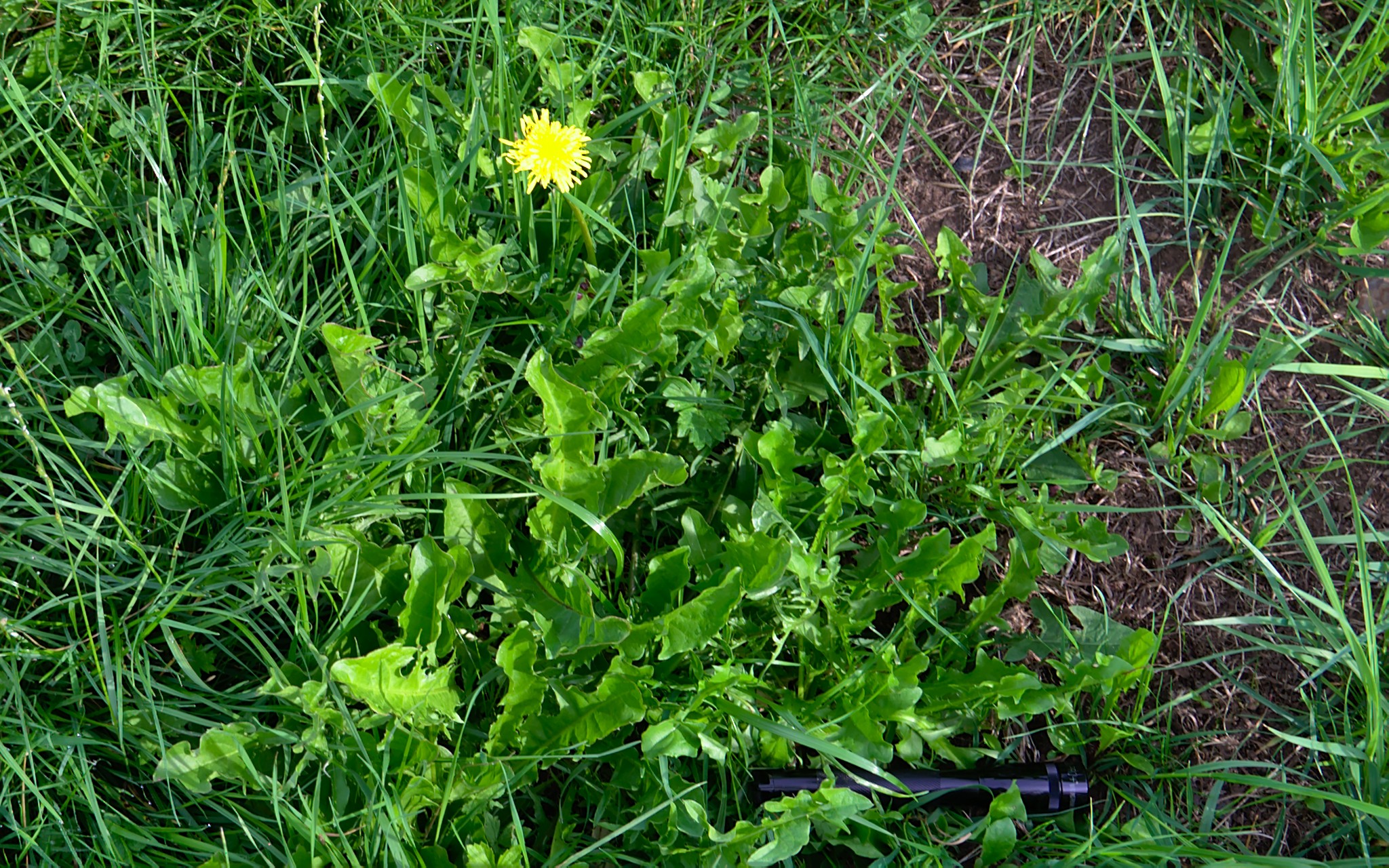

Supplement: S6 Data — This archive contains geo-referenced images used for the flower counting example in Fig 2 and the Python script implementing the pixel counting described in the caption to Fig 2. (TGZ) [file pcbi.1006141.s007.tgz › flowerExample/DSC_5885.jpg]

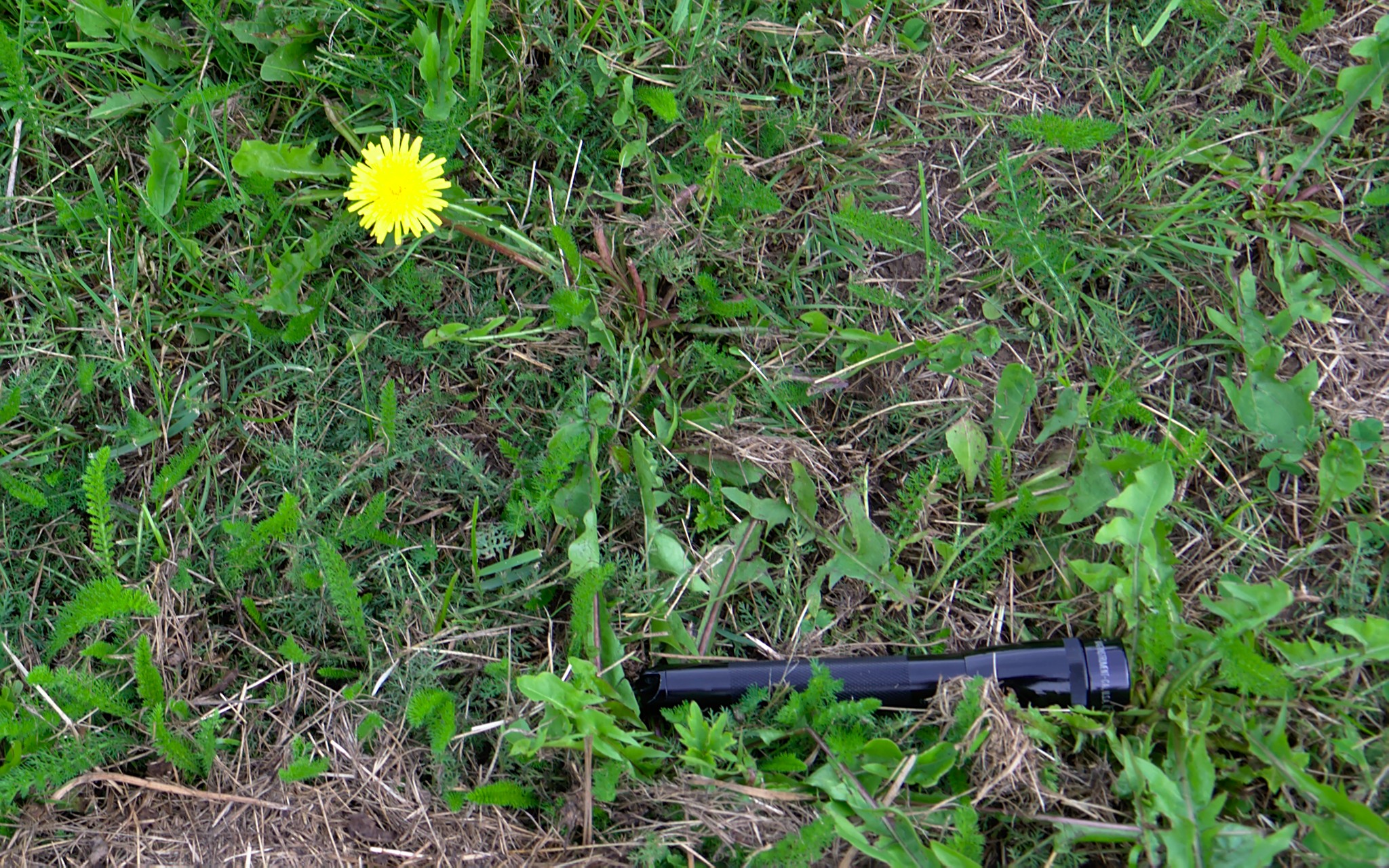

Supplement: S6 Data — This archive contains geo-referenced images used for the flower counting example in Fig 2 and the Python script implementing the pixel counting described in the caption to Fig 2. (TGZ) [file pcbi.1006141.s007.tgz › flowerExample/DSC_5886.jpg]

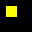

Supplement: S6 Data — This archive contains geo-referenced images used for the flower counting example in Fig 2 and the Python script implementing the pixel counting described in the caption to Fig 2. (TGZ) [file pcbi.1006141.s007.tgz › flowerExample/test_image.jpg]

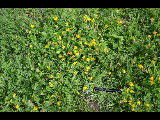

Supplement: S6 Data — This archive contains geo-referenced images used for the flower counting example in Fig 2 and the Python script implementing the pixel counting described in the caption to Fig 2. (TGZ) [file pcbi.1006141.s007.tgz › flowerExample/rawImages/DSC_5883.NEF]

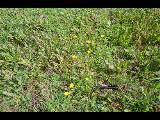

Supplement: S6 Data — This archive contains geo-referenced images used for the flower counting example in Fig 2 and the Python script implementing the pixel counting described in the caption to Fig 2. (TGZ) [file pcbi.1006141.s007.tgz › flowerExample/rawImages/DSC_5884.NEF]

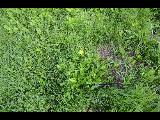

Supplement: S6 Data — This archive contains geo-referenced images used for the flower counting example in Fig 2 and the Python script implementing the pixel counting described in the caption to Fig 2. (TGZ) [file pcbi.1006141.s007.tgz › flowerExample/rawImages/DSC_5885.NEF]

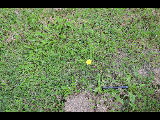

Supplement: S6 Data — This archive contains geo-referenced images used for the flower counting example in Fig 2 and the Python script implementing the pixel counting described in the caption to Fig 2. (TGZ) [file pcbi.1006141.s007.tgz › flowerExample/rawImages/DSC_5886.NEF]
